# Service Dogs for Veterans and Military Members With Posttraumatic Stress Disorder: A Nonrandomized Controlled Trial

**DOI:** 10.1001/jamanetworkopen.2024.14686

**Published:** 2024-06-04

**Authors:** Sarah C. Leighton, Kerri E. Rodriguez, Clare L. Jensen, Evan L. MacLean, Louanne W. Davis, Erin L. Ashbeck, Edward J. Bedrick, Marguerite E. O’Haire

**Affiliations:** 1 College of Veterinary Medicine, University of Arizona, Oro Valley; 2 Roudebush Veterans Affairs Medical Center, Indianapolis, Indiana; 3 Indiana University School of Medicine, Indianapolis; 4 Statistics Consulting Lab, The BIO5 Institute, University of Arizona, Tucson; 5 Department of Epidemiology and Biostatistics, College of Public Health, University of Arizona, Tucson

## Abstract

**Importance:**

Military members and veterans (hereafter, veterans) with posttraumatic stress disorder (PTSD) increasingly seek psychiatric service dogs as a complementary intervention, yet the effectiveness of service dogs is understudied.

**Objective:**

To estimate the associations between psychiatric service dog partnership and self-reported and clinician-rated PTSD symptom severity, depression, anxiety, and psychosocial functioning after 3 months of intervention among veterans.

**Design, Setting, and Participants:**

This nonrandomized controlled trial used standardized and validated assessment instruments completed by participants and administered by blinded clinicians. Recruitment, eligibility screening, and enrollment were conducted between August 2017 and December 2019. Veterans were recruited using the database of an accredited nonprofit service dog organization with constituents throughout the US. Participants were veterans with a PTSD diagnosis; they were allocated to either the intervention group (n = 81) or control group (n = 75). Outcome assessments were performed at baseline and at the 3-month follow-up. Data analyses were completed in October 2023.

**Interventions:**

Participants allocated to the intervention group received a psychiatric service dog for PTSD, whereas those allocated to the control group remained on the waiting list based on the date of application submitted to the service dog organization. Both groups had unrestricted access to usual care.

**Main Outcomes and Measures:**

The primary outcomes were PTSD symptom severity, depression, and anxiety after 3 months, and the secondary outcomes were psychosocial functioning, such as quality of life and social health. The self-reported PTSD Checklist for *Diagnostic and Statistical Manual of Mental Disorders*, Fifth Edition (*DSM-5*) was used to measure symptom severity, and the Clinician-Administered PTSD Scale for *DSM-5* (CAPS-5) was used to assess PTSD diagnosis (score range for both instruments: 0-80, with higher scores indicating greater PTSD symptoms).

**Results:**

The 156 participants included in the trial had a mean (SD) age of 37.6 (8.3) years and included 117 males (75%), 17 Black or African American individuals (11%), 30 Hispanic individuals (19%), and 117 White individuals (76%). Compared with the control group, the intervention group had significantly lower PTSD symptom severity based on the PTSD Checklist for *DSM-5* mean (SD) score (41.9 [16.9] vs 51.7 [16.1]; difference in means, −11.5 [95% CI, −16.2 to −6.6]; *P* < .001) and the CAPS-5 mean (SD) score (30.2 [10.2] vs 36.9 [10.2]; difference in means, −7.0 [95% CI, −10.8 to −4.5]; *P* < .001) at 3 months. The intervention group also had significantly lower depression scores (odds ratio [OR], 0.45 [95% CI, 0.23-0.86]; difference in means, −3.3 [95% CI, −6.8 to −0.6]), anxiety (OR, 0.25 [95% CI, 0.13-0.50]; difference in means, −4.4 [95% CI, −6.9 to −2.1]), and most areas of psychosocial functioning (eg, social isolation: OR, 0.34 [95% CI, 0.18-0.64]).

**Conclusions and Relevance:**

This nonrandomized controlled trial found that compared with usual care alone, partnership with a trained psychiatric service dog was associated with lower PTSD symptom severity and higher psychosocial functioning in veterans. Psychiatric service dogs may be an effective complementary intervention for military service–related PTSD.

**Trial Registration:**

ClinicalTrials.gov ID: NCT03245814

## Key Points

**Question:**

For military members and veterans with posttraumatic stress disorder (PTSD), is a partnership with a trained psychiatric service dog associated with lower PTSD symptom severity, lower anxiety, lower depression, and greater psychosocial functioning?

**Findings:**

In this nonrandomized controlled trial of 156 military members and veterans with PTSD, the addition of a service dog to usual care was associated with lower PTSD symptom severity, lower anxiety, and lower depression after 3 months of intervention.

**Meaning:**

Findings of this trial suggest that trained psychiatric service dogs may be an effective complement to usual care for military service–related PTSD.

## Teaser

This nonrandomized controlled trial examines subjectively and objectively assessed posttraumatic stress disorder (PTSD) symptom severity, anxiety, depression, and psychosocial functioning among US veterans with vs without a psychiatric service dog.

## Introduction

Posttraumatic stress disorder (PTSD) is a pressing concern for military members and veterans (hereafter, veterans), with an estimated prevalence of 23% among those with post-9/11 service.^1^ Posttraumatic stress disorder is characterized by symptoms of intrusion, avoidance of trauma reminders, adverse alterations in cognition and mood, and increased arousal and reactivity.^2^ By definition, disturbances must lead to clinically significant distress and/or impairment in areas of social, occupational, or other functioning.^2^ Posttraumatic stress disorder is associated with a number of comorbid conditions, including major depression and generalized anxiety disorder, and veterans are 1.5 times more likely to die by suicide than nonveteran adults.^3,4,5^

Currently, PTSD remains difficult to treat. Existing evidence-based treatments for PTSD are effective for some individuals, but uptake and retention are limited.^6^ Veterans are increasingly seeking out psychiatric service dogs (hereafter, service dogs) as complementary interventions. However, the effectiveness of service dogs remains understudied.^7^ Service dogs, referred to as *assistance dogs* internationally, are defined under US federal law as “dogs that are individually trained to do work or perform tasks for people with disabilities.”^8^ Preliminary evidence indicates that service dog partnerships are associated with meaningful improvements in self-reported PTSD symptoms for veterans with PTSD.^7^ However, only 1 clinical trial on their efficacy has been conducted to date,^9^ which compared emotional support dogs to service dogs, precluding conclusions about service dogs compared with usual care alone.^10^ Moreover, no studies of service dogs have used blinded or masked clinician ratings to evaluate PTSD severity outcomes.^7^ Therefore, a clinical trial using a no-dog comparison condition with blinded clinician ratings is needed to fill these gaps.

To our knowledge, the present trial represents the largest nationwide study to date to compare service dog partnerships with usual care alone and is the first National Institutes of Health–funded study to investigate service dog partnerships for military service–related PTSD. Prior publications have reported spouse,^11,12,13^ qualitative,^14^ biological,^15^ canine,^16^ and ecological momentary assessment^17^ data streams. The objective of this trial was to estimate the associations between service dog partnerships and self-reported and clinician-rated PTSD symptom severity, depression, anxiety, and psychosocial functioning after 3 months of intervention among veterans.

## Methods

### Trial Design and Participants

This prospective nonrandomized controlled trial compared veterans who received a trained service dog plus unrestricted access to usual care (hereafter, intervention group) with veterans who remained on a waiting list to receive a service dog and received unrestricted access to usual care (hereafter, control group). Participants were allocated to receive a service dog according to their position on the waiting list, which was ordered chronologically by application date, maintained by the service dog organization. The Purdue University Institutional Review Board and Institutional Animal Care and Use Committee approved this study; the study protocol is available in Supplement 1. Oral informed consent was obtained from each participant before enrollment and confirmed digitally prior to data collection. This trial was monitored by an independent Data and Safety Monitoring Board and was preregistered. We followed the Consolidated Standards of Reporting Trials (CONSORT) and Transparent Reporting of Evaluations with Nonrandomized Designs (TREND) reporting guidelines.^18,19^

Participants were recruited through the database of K9s For Warriors (K9FW), an Assistance Dogs International–accredited nonprofit service dog organization in the US, from August 2017 to December 2019. Data collection was completed in June 2020. Inclusion criteria were veterans who (1) applied for and were approved to receive a service dog from K9FW, including meeting K9FW’s eligibility criteria^20^; (2) were in military service on or after September 11, 2001; (3) had honorable discharge or current honorable service; (4) had current PTSD diagnosis assessed by blinded independent clinician evaluators; (5) had no conviction of any crimes against animals; and (6) were aged 18 years or older.

### Interventions

Participants in the intervention group received a trained service dog at no cost from K9FW, which acquires dogs primarily from animal shelters, owner relinquishments, and rescues (57%), after screening dogs for health and temperament.^16^ Breeds were predominantly mixed (59%), and the most common pure breed was a Labrador retriever (22%).^16^ Service dogs received at least 60 hours of professional training and passed a final obedience and specialized skill proficiency test. Specialized PTSD-related skills included interrupt or alert to anxiety, calm or comfort anxiety, block (create space), cover (watch back), and make a friend (social greeting).^21^

Veterans were partnered with service dogs during a 3-week, onsite, group class (6-12 veterans) at the K9FW campus in Ponte Vedra, Florida. The curriculum included 40 hours per week of instruction in service dog care, training, and interaction (≥10 hours in public settings); a training manual; and written and hands-on assessments. Veteran–service dog dyads passed the Assistance Dogs International Public Access Test, a standardized assessment intended to demonstrate control and safety in public. After training and service dog partnership, K9FW maintained contact and provided support to veterans at regular intervals for the entire duration of the partnership. Intervention delivery and enactment was assessed using the Fidelity Checklist for Research on Assistance Dogs (eTable 1 in Supplement 2).

Participants in the control group were recruited from the K9FW waiting list. All participants had unrestricted access to usual care.

### Outcomes

Prespecified outcomes were assessed at baseline (prior to service dog allocation in the intervention group) and at follow-up (approximately 3 months after the completion of baseline). Service dog allocation in the intervention group took place approximately 5 days after the baseline assessment. Demographic characteristics, including age, race, ethnicity, gender identity, relationship status, disability status, and socioeconomic status (income adequacy), were self-reported at baseline. Race and ethnicity data were assessed because studies have found substantial race and ethnicity–based differences in PTSD symptom endorsement,^22^ treatment initiation,^23^ and treatment administration.^24^

Primary outcomes were PTSD symptom severity, depression, and anxiety after 3 months. Symptom severity was measured with the self-reported PTSD Checklist for *DSM-5* (*Diagnostic and Statistical Manual of Mental Disorders* [Fifth Edition]) (PCL-5; α = 0.96).^25^ Blinded, independent assessment was conducted with the Clinician-Administered PTSD Scale for *DSM-5* (CAPS-5; α = 0.73-0.95)^26,27^; CAPS-5 was used to assess PTSD diagnosis. Both PCL-5 and CAPS-5 had a score range of 0 to 80, with higher scores indicating greater PTSD symptoms.

Including both subjective (self-report) and objective (blinded clinician assessment) measures of PTSD symptoms strengthens the reliability of these findings and reflects clinical practice to help inform evidence-based practices. The clinician raters were blinded to the study topic (service dogs), design, timing (baseline or follow-up), and condition allocation (intervention or control). The CAPS-5 raters were clinical psychology doctoral students trained by an experienced US Department of Veterans Affairs (VA) clinician (L.W.D.). Both the PCL-5 and the CAPS-5 were conducted with reference to an index event (ie, the worst or most salient currently distressing event), which was identified using the Life Events Checklist for *DSM-5*.^28^ Depression was measured with the National Institutes of Health Patient-Reported Outcomes Measurement Information System (PROMIS) Short Form version 1.0 Depression (Cronbach α = 0.95-0.97; score range: 38-81, with higher scores indicating greater depression). Anxiety was measured with the PROMIS Anxiety (Cronbach α = 0.98; score range: 37-83, with higher scores indicating greater anxiety).^29,30^

The secondary outcomes were psychosocial functioning, such as quality of life and social health. Social health was measured with the PROMIS Short Form version 2.0 Ability to Participate in Social Roles and Activities (score range: 25-65, with higher scores indicating higher social activity), Social Isolation (score range: 33-76, with lower scores indicating less isolation), and Companionship (score range: 24-64, with higher scores indicating higher companionship).^29^ Quality of life was measured with the Bradburn Scale of Psychological Well-being (BSPW; Cronbach α = 0.85; score range: −5 to 5, with higher scores indicating better well-being),^31^ the Satisfaction With Life Scale (SWLS; score range: 3-35, with higher scores indicating higher satisfaction),^32^ the 10-Item Connor-Davidson Resilience Scale (CD-RISC-10; Cronbach α = 0.89; score range: 0-40, with higher scores indicating greater resilience),^33^ the Veterans RAND 12-Item Health Survey Mental Component Score (VR-12 MCS; score range: 0-100, with higher scores indicating better mental health),^34^ and PROMIS Short Form version 1.0 for the Anger domain (Cronbach α = 0.97; score range: 32-82, with lower scores indicating less anger).^30^

Suicidality was monitored, and data were captured in descriptive format. Suicidality was measured using the Columbia-Suicide Severity Rating Scale (C-SSRS; Cronbach α = 0.73-0.95)^27,35^ and the 9-item Patient Health Questionnaire (PHQ-9; Cronbach α = 0.89; score range: 0-27, with lower scores indicating less depression).^36,37^ A validated action protocol was implemented to connect participants with information and resources in the event of high suicide risk. Exploratory outcomes included PCL-5 and CAPS-5 subscales, specifically: intrusion, avoidance, cognition and mood, and arousal and reactivity.^25,26^

### Adverse Events and Sample Size

Adverse events were collected from passive surveillance, typically due to events that affected study participation.^38^ The minimum sample size was planned to be 50 participants per group to allow for detection of a moderate effect size (Cohen *d* = 0.40), with the probability of a type I error of .05 and power of 0.80. Using a conservative 22% noncompletion rate based on reports from clinical trials among veterans with PTSD, we planned to enroll at least 150 veterans.

### Statistical Analysis

We fit multivariable ordinal cumulative probability models with a logit link for primary, secondary, and other outcomes.^39,40^ Models included a treatment variable for the intervention vs control groups as well as prespecified covariates assessed at baseline, including age, race, ethnicity, and gender identity as well as military sexual trauma, traumatic brain injury (assessed with the 3-item Brief Traumatic Brain Injury Survey^41^), concurrent evidence-based PTSD treatment (assessed with a shortened version of the American Legion Survey of Patient Healthcare Experiences and defined according to VA and Department of Defense clinical practice guidelines^42,43^), pet dog ownership, and the baseline score for the modeled outcome. Ordinal cumulative probability models were selected because they incorporate the order information of the response variable, do not assume data are interval or ratio scaled,^44^ are well suited for modeling responses that are skewed with floor or ceiling effects, and are appropriate for discrete ordinal distributions and continuous responses.^45,46^ Since the conditional cumulative distribution function is modeled directly, these models also enable the estimation of exceedance probabilities of interest with greater efficiency than dichotomization.^47^

Multiple imputation was used to account for uncertainty in missing covariate values and missing outcomes.^48,49^ We reported estimated odds ratios (ORs), differences in means, and differences in exceedance probabilities (absolute risk reduction) between the intervention group and the control group with bootstrapped percentile nonparametric 95% CIs.^50,51^

Since the association between service dog partnership and PTSD severity at follow-up could differ based on the severity of PTSD at baseline, we included an interaction between intervention (service dog vs waiting list) and baseline PTSD severity score and conducted a likelihood ratio test for the interaction term.

As a sensitivity analysis, we fit linear regression models to estimate the differences in means between the groups, with the same planned covariates and multiple imputation approach used in the ordinal cumulative probability models. We estimated a standardized effect size, Cohen *d*, in a sample that included only participants with follow-up data.

Two-sided *P* < .05 indicated statistical significance. Analyses were completed in October 2023 using R version 4.3.0 (R Project for Statistical Computing).^52^

## Results

Of the 200 veterans assessed for eligibility, 170 were deemed eligible, consented to participate, and enrolled (Figure). Among 91 participants allocated to the intervention group, 81 received a service dog, whereas 75 of 79 participants allocated to the control group remained on the waiting list. Thus, among 170 participants enrolled in the study, 14 were excluded from analysis because they did not receive the allocated intervention, leaving an analysis sample of 156 participants. The intervention dropout proportion was 0.10: of the 81 participants who received a service dog, 8 returned the service dog. Among 156 participants who received the allocated intervention, 143 (92%) completed the follow-up PCL-5 assessment and 135 (87%) completed the follow-up CAPS-5.

**Figure.**
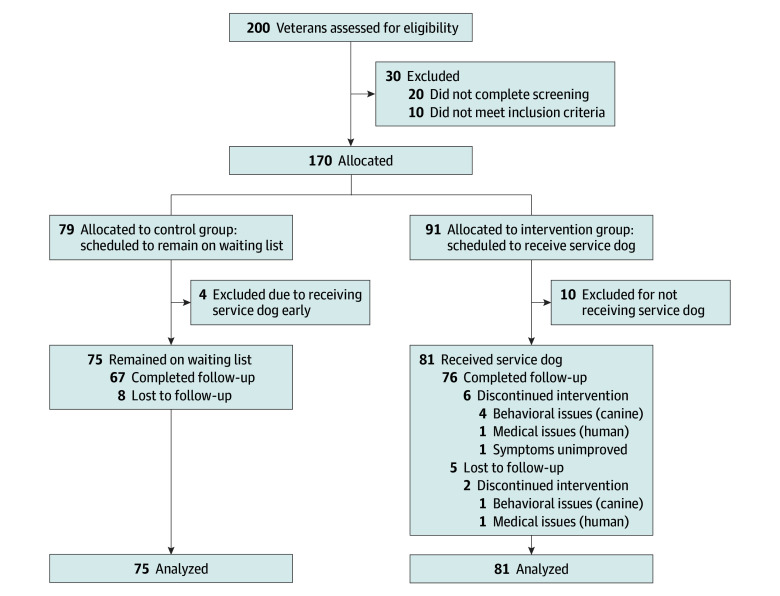
Participant Flow Diagram

The mean (SD) age of participants was 37.6 (8.3) years. Among participants, 39 (25%) self-reported as female, 117 (75%) as male, 2 (1%) as Asian, 17 (11%) as Black or African American, 30 (19%) as Hispanic or Latino individuals; 3 (2%) as Native Hawaiian or Other Pacific Islander, 117 (76%) as White, and 8 participants (5%) identified as having more than 1 race. Sixty-four households (42%) had pet dogs at baseline. Full demographic and clinical data are presented in Table 1.

**Table 1.** Baseline Demographic and Clinical Characteristics

	Intervention group, No./total No. (%)	Control group, No./total No. (%)	No./total No. (%)
Demographic characteristics			
Age, mean (SD), y	37.0 (8.2)^a^	38.2 (8.5)^b^	37.6 (8.3)^c^
Gender identity			
Female	17/81 (21)	22/75 (29)	39/156 (25)
Male	64/81 (79)	53/75 (71)	117/156 (75)
Race^d^			
American Indian or Alaska Native	0/79	0/74	0/153
Asian	0/79	2/74 (3)	2/153 (1)
Black or African American	8/79 (10)	9/74 (12)	17/153 (11)
Native Hawaiian or Other Pacific Islander	2/79 (3)	1/74 (1)	3/153 (2)
White	62/79 (78)	55/74 (74)	117/153 (76)
More than 1 race	3/79 (4)	5/74 (7)	8/153 (5)
Prefer not to say	4/79 (5)	2/74 (3)	6/153 (4)
Ethnicity^d^			
Hispanic or Latino	15/81 (19)	15/75 (20)	30/156 (19)
Not Hispanic or Latino	62/81 (77)	59/75 (79)	121/156 (78)
Prefer not to say	4/81 (5)	1/75 (1)	5/156 (3)
Relationship status			
Divorced	12/81 (15)	10/75 (13)	22/156 (14)
Living with significant other	3/81 (4)	4/75 (5)	7/156 (5)
Married	45/81 (56)	53/75 (71)	98/156 (63)
Single	14/81 (17)	6/75 (8)	20/156 (13)
Separated	7/81 (9)	2/75 (3)	9/156 (6)
Widowed	0/81	0/75	0/156
Educational level			
Some high school	0/81	0/74	0/155
High school diploma or GED	7/81 (9)	4/74 (5)	11/155 (7)
Some college	32/81 (40)	22/74 (30)	54/155 (35)
2-y College degree	14/81 (17)	10/74 (14)	24/155 (15)
4-y College degree	17/81 (21)	21/74 (28)	38/155 (25)
Postgraduate degree	11/81 (14)	17/74 (23)	28/155 (18)
Employment status			
Employed	28/81 (35)	27/73 (37)	55/154 (36)
Homemaker	3/81 (4)	1/73 (1)	4/154 (3)
Out of work	9/81 (11)	4/73 (6)	13/154 (8)
Retired	16/81 (20)	10/73 (14)	26/154 (17)
Student	6/81 (7)	9/73 (12)	15/154 (10)
Unable to work for health or disability reasons	18/81 (22)	22/73 (30)	40/154 (26)
Volunteer	1/81 (1)	0/73	1/154 (1)
Military characteristics			
Deployed	71/80 (89)	59/73 (81)	130/153 (85)
Military branch^e^			
Air Force	5/77 (7)	6/57 (11)	11/134 (8)
Army	49/77 (64)	30/57 (53)	79/134 (59)
Coast Guard	1/77 (1)	2/57 (4)	3/134 (2)
Marine Corps	15/77 (19)	7/57 (12)	22/134 (16)
National Guard	7/77 (9)	3/57 (5)	10/134 (8)
Navy	5/77 (7)	13/57 (23)	18/134 (13)
Household characteristics			
No. of household members, median (IQR)	3 (2-4)^f^	3 (2-5)^g^	3 (2-4)^h^
No. of children, median (IQR)	1 (0-2)^f^	1 (0-3)^g^	1 (0-2)^h^
Pet dog ownership	32/80 (40)	32/73 (44)	64/153 (42)
Income			
Comfortable	27/80 (34)	35/73 (48)	62/153 (41)
Just enough to make ends meet	46/80 (58)	33/73 (45)	79/153 (52)
Not enough to make ends meet	7/80 (9)	5/73 (7)	12/153 (8)
Clinical characteristics			
Comorbid conditions			
Deployment-related MST	19/80 (24)	18/73 (25)	37/153 (24)
Deployment-related TBI	35/80 (44)	36/73 (49)	71/153 (46)
Concurrent evidence-based treatment	23/80 (29)	16/73 (22)	39/153 (25)
CPT	14/80 (18)	12/73 (16)	26/153 (17)
EMDR therapy	3/80 (4)	5/73 (7)	8/153 (5)
PE therapy	13/80 (16)	2/73 (3)	15/153 (10)
Suicidal behavior: C-SSRS lifetime	33/80 (41)	41/75 (55)	74/155 (48)

Abbreviations: C-SSRS, Columbia-Suicide Severity Rating Scale; CPT, Cognitive Processing Therapy; EMDR, Eye Movement Desensitization and Reprocessing; GED, General Educational Development; MST, military sexual trauma; PE, prolonged exposure; TBI, traumatic brain injury.

^a^ Total No. in the intervention group was 81.

^b^ Total No. in the control group was 75.

^c^ Total No. overall was 156.

^d^ Race and ethnicity were self-reported by participants.

^e^ Percentages may exceed 100%. Some participants served with multiple branches.

^f^ Total No. in the intervention group was 79.

^g^ Total No. in the control group was 73.

^h^ Total No. overall was 152.

### PTSD, Depression, and Anxiety

Participants in the intervention group reported significantly lower PTSD symptom severity after 3 months compared with participants in the control group, based on the PCL-5 (OR, 0.22 [95% CI, 0.12-0.42]; mean [SD] score, 41.9 [16.9] vs 51.7 [16.1]; difference in means, −11.5 [95% CI, −16.2 to −6.6]) and the CAPS-5 (OR, 0.21 [95% CI, 0.11-0.40]; mean [SD] score, 30.2 [10.2] vs 36.9 [10.2]; difference in means, −7.0 [95% CI, −10.8 to −4.5]) outcomes (Table 2; eFigure in Supplement 2). There was also a significant difference in the odds of meeting CAPS-5 diagnostic criteria for PTSD (OR, 0.34; 95% CI, 0.12-0.97), with 75% (51) of the intervention group vs 85% (56) of the control group receiving a PTSD diagnosis at follow-up. In the current sample using blinded CAPS-5 raters, interrater reliability was strong (diagnosis: Gwet AC1 = 0.93 [95% CI, 0.85-1.00]^53^; severity: intraclass correlation coefficient (2,1) = 0.95 [95% CI, 0.94-0.98]).

**Table 2.** Association Between Service Dog Partnership and Primary and Secondary Outcomes at 3-Month Follow-Up

Outcome	Mean (SD) score				Group comparison at 3 mo^a^		
	Intervention group		Control group		Difference in group means (95% CI)	OR (95% CI)	*P* value
	Baseline (n = 81)	3 mo (n = 76)^b^	Baseline (n = 75)	3 mo (n = 67)^b^			
Primary outcomes							
PTSD							
PCL-5	57.0 (11.3)	41.9 (16.9)	55.7 (14.3)	51.7 (16.1)	−11.5 (−16.2 to −6.6)	0.22 (0.12 to 0.42)	<.001
CAPS-5	42.0 (7.6)	30.2 (10.2)	40.0 (7.0)	36.9 (10.2)	−7.0 (−10.8 to −4.5)	0.21 (0.11 to 0.40)	<.001
Depression and anxiety							
PROMIS Depression^c^	64.9 (7.8)	58.9 (9.5)	62.7 (8.4)	61.4 (8.0)	−3.3 (−6.8 to −0.6)	0.45 (0.23 to 0.86)	.02
PROMIS Anxiety^c^	68.2 (5.8)	62.1 (7.1)	66.5 (5.5)	66.0 (5.4)	−4.4 (−6.9 to −2.1)	0.25 (0.13 to 0.50)	<.001
Secondary outcomes							
Social health							
PROMIS Social Isolation^c^	65.3 (8.2)	60.1 (10.8)	62.7 (8.8)	62.8 (8.6)	−4.3 (−7.4 to −1.6)	0.34 (0.18 to 0.64)	.001
PROMIS Companionship^c^	44.9 (10.7)	48.5 (10.1)	46.9 (9.5)	45.2 (9.3)	3.9 (1.3 to 7.0)	2.83 (1.47 to 5.45)	.003
PROMIS Social Activities^c^	50.8 (8.2)	45.2 (8.3)	50.1 (6.3)	49.6 (6.3)	−4.9 (−7.1 to −2.6)	0.24 (0.12 to 0.48)	<.001
Quality of life							
BSPW	−2.7 (1.8)	−0.6 (2.7)	−2.2 (1.9)	−2.2 (2.2)	1.7 (0.9 to 2.5)	4.49 (2.28 to 8.83)	<.001
Positive affect	1.4 (1.5)	2.5 (1.7)	1.5 (1.5)	1.6 (1.6)	1.0 (0.4 to 1.6)	3.15 (1.60 to 6.23)	.002
Negative affect	4.0 (1.1)	3.0 (1.4)	3.8 (1.1)	3.8 (1.0)	−0.8 (−1.3 to −0.5)	0.21 (0.11 to 0.42)	<.001
SWLS	13.3 (6.3)	19.7 (7.0)	13.8 (6.6)	15.3 (6.9)	4.1 (2.0 to 6.1)	3.73 (1.88 to 7.40)	<.001
CD-RISC-10	17.5 (7.5)	21.7 (6.3)	20.8 (7.2)	20.8 (7.7)	2.5 (0.5 to 4.4)	2.33 (1.22 to 4.47)	.02
VR-12 MCS	26.0 (11.3)	36.3 (10.7)	28.2 (9.9)	29.1 (10.4)	7.4 (4.1 to 11.7)	3.84 (2.00 to 7.38)	<.001
PROMIS Anger^c^	68.7 (9.1)	61.2 (10.5)	64.9 (8.7)	64.1 (10.0)	−4.8 (−8.6 to −1.1)	0.39 (0.20 to 0.75)	.009

Abbreviations: BSPW, Bradburn Scale of Psychological Well-being (score range: −5 to 5, with higher scores indicating better well-being; subscales range: 0-5, with higher scores indicating higher positive or negative affect); CAPS-5, Clinician-Administered PTSD Scale for *DSM-5* (score range: 0-80, with higher scores indicating greater PTSD symptoms); CD-RISC-10, 10-Item Connor-Davidson Resilience Scale (score range: 0-40, with higher scores indicating greater resilience); *DSM-5, Diagnostic and Statistical Manual of Mental Disorders* (Fifth Edition); OR, odds ratio; PCL-5, PTSD Checklist for *DSM-5* (score range: 0-80, with higher scores indicating greater PTSD symptoms); PROMIS, Patient-Reported Outcomes Measurement Information System; PTSD, posttraumatic stress disorder; SWLS, Satisfaction With Life Scale (score range: 3-35, with higher scores indicating higher satisfaction); VR-12 MCS, Veterans RAND 12-Item Health Survey Mental Component Score (score range: 0-100, with higher scores indicating better mental health).

^a^ Differences between group means and ORs included all 156 participants and were estimated from an ordinal cumulative probability model after multiple imputation of missing outcome scores and missing covariate values. Model covariates included the baseline score (restricted cubic splines), age, gender identity, race (White compared with Black, Indigenous, and other minoritized groups), Hispanic ethnicity, pet dog, military sexual trauma, traumatic brain injury, and concurrent evidence-based treatment reported at baseline. *P* values were from a likelihood ratio test.

^b^ Mean (SD) values were calculated based on participants with available data. The number of participants assessed for PCL-5 at follow-up in the intervention and control groups was 76 and 67, respectively; for CAPS-5 follow-up, there were 69 and 66 participants. For all other outcomes, there were 68 and 65 participants assessed at follow-up.

^c^ PROMIS Depression score range: 38 to 81, with higher scores indicating greater depression (minimally important difference: ≥3 points); Anxiety score range: 37 to 83, with higher scores indicating greater anxiety (minimally important difference: ≥3 points); Social Isolation score range: 33 to 76, with lower scores indicating less isolation; Companionship score range: 24 to 64, with higher scores indicating higher companionship; Social Activities score range: 25 to 65, with higher scores indicating higher social activity; Anger score range: 32 to 82, with lower scores indicating less anger.

PROMIS Depression scores were significantly lower after 3 months for participants in the intervention group compared with the control group (OR, 0.45 [95% CI, 0.23-0.86]; difference in means, −3.3 [95% CI, −6.8 to −0.6]). Participants in the intervention group also had lower probability of at least mild depression (PROMIS Depression score ≥55 at 3 months; 0.76 vs 0.88; absolute risk difference, −0.12 [95% CI, −0.29 to −0.02]) (eFigure in Supplement 2).

Participants in the intervention group had significantly lower PROMIS Anxiety scores after 3 months (OR, 0.25 [95% CI, 0.13-0.50]; difference in means, −4.4 [95% CI, −6.9 to −2.1]), and lower probability of generalized anxiety disorder (PROMIS Anxiety score of ≥62.3; 0.48 vs 0.78; absolute risk difference, −0.30 [95% CI, −0.48 to −0.12]) (eFigure in Supplement 2).

### Psychosocial Functioning

Secondary outcomes analysis indicated better social health in the intervention group in terms of less social isolation (PROMIS Social Isolation: OR, 0.34; 95% CI, 0.18-0.64) and higher companionship (PROMIS Companionship: OR, 2.83; 95% CI, 1.47-5.45) compared with the control group. However, we did find significantly lower social activity for participants in the intervention group vs the control group (PROMIS Social Activities: OR, 0.24; 95% CI, 0.12-0.48). Analysis also indicated higher quality of life in the intervention group across all measures, including better well-being (BSPW: OR, 4.49; 95% CI, 2.28-8.83), greater life satisfaction (SWLS: OR, 3.73; 95% CI, 1.88-7.40), greater resilience (CD-RISC-10: OR, 2.33; 95% CI, 1.22-4.47), better mental health (VR-12 MCS: OR, 3.84; 95% CI, 2.00-7.38), and less anger (PROMIS Anger: OR, 0.39; 95% CI, 0.20-0.75) (Table 2).

Suicidality was present in the study sample from baseline to follow-up (C-SSRS item 1: from 44 participants [55%] to 26 participants [35%] in the intervention group vs from 35 [47%] to 31 [46%] in the control group; PHQ-9 item 9: from 38 [48%] to 21 [31%] in the intervention group vs from 34 [47%] to 28 [43%] in the control group). Full description and C-SSRS and PHQ-9 results are provided in eAppendix and eTables 5 to 7 in Supplement 2.

### Exploratory and Sensitivity Analyses

Analyses of PCL-5 and CAPS-5 subscales suggested that compared with being on the waiting list, a service dog partnership was associated with lower PTSD symptom severity in all domains based on the subscales of the PCL-5 and CAPS-5, including intrusion, avoidance, cognition and mood, and arousal and reactivity (Table 3). The interaction between intervention and baseline severity score was not significant for any of the 4 primary outcome measures (PCL-5, CAPS-5, PROMIS Depression, and PROMIS Anxiety) based on likelihood ratio tests for the interaction terms.

**Table 3.** Association Between Service Dog Partnership and PCL-5 and CAPS-5 Subscales at 3-Month Follow-Up

Outcome	Mean (SD) score				Group comparison at 3 mo^a^		
	Intervention group		Control group		Difference in group means (95% CI)	OR (95% CI)	*P* value
	Baseline (n = 81)	3 mo (n = 76)^b^	Baseline (n = 75)	3 mo (n = 67)^b^			
**Other outcomes**							
PTSD: PCL-5 subscales							
Intrusion^c^	13.7 (3.8)	10.4 (4.8)	13.8 (4.3)	12.6 (4.7)	−2.2 (−3.6 to −0.8)	0.37 (0.20 to 0.70)	.003
Avoidance^c^	6.5 (1.7)	4.8 (2.3)	6.1 (1.9)	6.1 (2.1)	−1.6 (−2.3 to −0.9)	0.24 (0.13 to 0.46)	<.001
Cognition and Mood^c^	19.5 (5.2)	14.6 (6.7)	19.3 (6.0)	17.9 (6.9)	−3.4 (−5.5 to −1.3)	0.35 (0.19 to 0.64)	<.001
Arousal and Reactivity^c^	17.4 (3.6)	12.6 (5.2)	16.5 (4.3)	15.2 (5.1)	−3.5 (−5.0 to −1.9)	0.25 (0.13 to 0.46)	<.001
PTSD: CAPS-5 subscales							
Intrusion^c^	9.8 (2.9)	6.8 (3.1)	9.5 (2.6)	8.4 (3.4)	−1.5 (−2.5 to −0.5)	0.39 (0.21 to 0.71)	.004
Avoidance^c^	5.0 (1.4)	3.6 (1.9)	5.2 (1.1)	4.9 (1.5)	−1.3 (−1.9 to −0.7)	0.23 (0.12 to 0.46)	<.001
Cognition and Mood^c^	15.1 (4.1)	11.5 (5.3)	14.5 (3.3)	13.5 (4.4)	−2.3 (−3.8 to −0.8)	0.34 (0.17 to 0.68)	.003
Arousal and Reactivity^c^	12.0 (2.7)	8.3 (3.3)	10.8 (2.7)	10.1 (3.3)	−2.2 (−3.5 to −1.2)	0.25 (0.13 to 0.49)	<.001

Abbreviations: CAPS-5, Clinician-Administered PTSD Scale for *DSM-5*; *DSM-5, Diagnostic and Statistical Manual of Mental Disorders* (Fifth Edition); OR, odds ratio; PCL-5, PTSD Checklist for *DSM-5* (score range: 0-80, with higher scores indicating greater PTSD symptoms); PTSD, posttraumatic stress disorder.

^a^ Differences between group means and ORs included all 156 participants and were estimated from an ordinal cumulative probability model after multiple imputation of missing outcome scores and missing covariate values. Model covariates included the baseline score (restricted cubic splines), age, gender identity, race (White compared with Black, Indigenous, and other minoritized groups), Hispanic ethnicity, pet dog, military sexual trauma, traumatic brain injury, and concurrent evidence–based treatment reported at baseline. *P* values were from a likelihood ratio test.

^b^ Mean (SD) values were calculated using participants with available data. The number of participants assessed for PCL-5 at follow-up in the intervention and control groups were 76 and 67, respectively. For CAPS-5 follow-up, there were 69 and 66 participants.

^c^ PCL-5 and CAPS-5 Intrusion score range: 0 to 20; Avoidance score range: 0 to 8; Cognition and Mood score range: 0 to 28; Arousal and Reactivity score range: 0 to 24. Higher subscale scores indicate greater symptoms within that cluster.

We used linear regression as a sensitivity analysis and found similar results, and the estimated standardized effect sizes (Cohen *d*) are reported in eTable 2 in Supplement 2. In a per-protocol analysis for the primary outcomes, we further restricted the sample by excluding 8 participants who returned their service dog and found similar results (eTable 3 in Supplement 2). Participants reported a total of 11 adverse events (eTable 4 in Supplement 2).

## Discussion

Compared with the control group, veterans in the intervention group had significantly lower self-reported and clinician-rated PTSD symptom severity, significantly lower anxiety and depression, significantly higher quality of life, and mixed social health outcomes (less isolation and activity participation, and more companionship). Overall, most findings supported favorable outcomes for veterans who received service dogs.

This trial’s findings of lower PTSD symptom severity are consistent with results of previous studies of service dogs for veterans with PTSD^7,54^ while adding the first blinded ratings to confirm this finding clinically. These results are notable given the relatively short follow-up period (3 months) compared with the typical service dog partnership length (≥8 years). Although specific mechanisms for potential benefits remain unknown, prior research has identified an association between the service dog’s trained tasks and the presence of psychosocial functioning as well as potential stress hormone pathways via the cortisol awakening response in veterans.^15,16,17,21,54^

Service dog partnerships were also associated with a loss of clinician PTSD diagnosis.^55^ Given that participants also had unrestricted access to usual care, study findings support suggestions from prior research that service dog partnerships should take place in combination with other evidence-based care.^7,56^

The intervention dropout proportion for this study (0.10) was substantially lower than the reported dropout for both trauma-focused (0.27; 95% CI, 0.21-0.34) and nontrauma-focused treatments (0.16; 95% CI, 0.12-0.21).^6^ Retention in effective, evidence-based treatments is a challenge for veterans with PTSD. Therefore, research such as the present trial is critical to identify and examine promising complementary interventions, including service dog partnership, that expand the range of options available to veterans with a wide variety of needs. Furthermore, it is essential for future research to examine the combination of a service dog intervention and existing evidence-based therapy to ascertain whether the combination can achieve PTSD symptom reduction and adherence to treatment.

Based on standardized effect size, service dog partnership was associated with medium to large improvements in most areas of psychosocial functioning, including quality of life, well-being, and life satisfaction. Decreases in social participation after service dog partnership could be attributed to adverse experiences (or anticipation of adverse experiences), such as access denials and stigma when accompanied by a service dog in public.^17,57^

### Limitations

This trial has several limitations. First, it used nonrandom allocation of treatment. Participants received service dogs based on their position on the waiting list, which was determined by their application date. However, veterans on the waiting list were similar to participants who received a service dog, as suggested by the distributions of baseline characteristics, and our analyses included planned adjustments for baseline characteristics believed to be most relevant. Second, CAPS-5 raters were blinded to the trial topic, assessment timing, and allocation group, but other outcomes were limited by self-reporting biases. Third, the findings may not be generalizable to veterans with PTSD who do not seek out service dogs. Fourth, service dogs were trained by a single organization; fidelity, adherence, and dropout rates may be different across service dog organizations.

## Conclusions

Compared with usual care alone, partnership with a trained psychiatric service dog was associated with lower PTSD symptom severity and better psychosocial functioning for US military members and veterans after only 3 months of this intervention. Based on standardized self-reported and clinician-assessed symptom severity, service dog partnership may serve as an effective complementary intervention for military service–related PTSD.

## Notes

Supplement 1.
Trial Protocol

Supplement 2. **eTable 1.** Fidelity Checklist for Research on Assistance Dogs (F-RAD) **eTable 2.** Cohen’s *d* and Linear Regression Sensitivity Analysis **eTable 3.** Per Protocol Analysis: Primary Outcomes at 3 Months **eFigure.** Primary Outcomes: Exceedance Probability Distributions, Conditional on Service Dog vs Control Group **eTable 4.** Self-Reported Adverse Events (AE) and Serious Adverse Events (SAE) **eAppendix.** Columbia Suicide Severity Rating Scale (C-SSRS) and Patient Health Questionnaire (PHQ-9) Data **eTable 5.** Participant C-SSRS Data at Baseline and 3-Month Follow-Up **eTable 6.** Participant PHQ-9 Item 9 Data at Baseline and 3-Month Follow-Up **eTable 7.** PHQ-9 Depression at 3 Months
eReferences

Supplement 3.
Data Sharing Statement
