# Supplementary material for: Service Dogs for Veterans and Military Members With Posttraumatic Stress Disorder: A Nonrandomized Controlled Trial
Source: JAMA Netw Open. 2024 Jun 4;7(6):e2414686. doi: 10.1001/jamanetworkopen.2024.14686 (PMC11151141; doi:10.1001/jamanetworkopen.2024.14686)
Supplement: Supplement 1. — Trial Protocol [file jamanetwopen-e2414686-s001.pdf]

Supplement 1. Research Protocol

Title: Quantifying the Efficacy and Role of Service Dogs for Military Veterans With PTSD
Sponsor: Dr. Marguerite E. O'Haire
ClinicalTrials.gov ID: NCT03245814

Brief Summary

The purpose of this study is to quantify the therapeutic efficacy and role of trained service dogs on socio-emotional functioning among military veterans with posttraumatic stress disorder (PTSD).

Detailed Description

The long-term research goal is to evaluate the safety and efficacy of service dogs as a complementary intervention to enhance biopsychosocial functioning in special populations. The objective is to conduct a methodologically rigorous trial to quantify the therapeutic efficacy of service dogs on clinically important outcomes for veterans with PTSD. Based on preliminary findings and qualitative reports, the central hypothesis is that military veterans with PTSD who are provided service dogs will experience reduced PTSD symptoms related to socio-emotional functioning and arousal modulation. The rationale for this research is that its successful completion will provide an evidence-based demonstration of the efficacy and role of an increasingly used yet poorly tested complementary intervention. The completion of this project is expected to establish an initial demonstration of the therapeutic efficacy of service dogs in this population, as well as possible mechanisms of action via specific biological pathways and human-canine interaction profiles.
Conditions: Posttraumatic Stress Disorder (PTSD)
Intervention / Treatment: Behavioral: Service Dog

Funding

This work was supported by the Eunice Kennedy Shriver National Institute of Child Health and Human Development (NICHD) and the National Center for Complementary and Integrative Health (NCCIH) of the National Institutes of Health (NIH) under award number R21HD091896, Merrick PetCare and the Dogtopia Foundation. The content is solely the responsibility of the authors and does not necessarily represent the official views of the National Institutes of Health.

Participant Criteria

Eligibility Criteria

Inclusion criteria for veterans into the study will include:

- Applied for and approved to receive a dog from K9s For Warriors
- Military service on or after September 11, 2001
- Honorable discharge or current honorable service
- Diagnosis of PTSD
- No conviction of any crimes against animals.
- 18 Years and older

Study Plan

Study Design

Primary Purpose: Supportive Care
Allocation: Non-Randomized
Interventional Model: Parallel Assignment
Masking: Single (Outcomes Assessor)
Masking Description: Clinician Administered PTSD Scale for DSM-V (CAPS-5) assessments will be completed via telephone by a trained CAPS-5 rater who will be masked to the intervention and treatment arm of each participant.

Arms and Interventions

Participant / Group Arm	Intervention / Treatment
Experimental: Service Dog<br>Participants in the service dog arm will receive unrestricted, non-study usual care, in addition to a trained service dog.	Behavioral: Service Dog<br>A service dog trained to perform tasks that are specific to PTSD
No Intervention: Waitlist Control<br>Participants in the control arm will receive unrestricted, non-study usual care, while on the waitlist for a service dog.	

Outcome Measures

Primary

Outcome Measure	Measure Description	Time Frame
**PTSD severity and symptoms via self-report**	Posttraumatic Stress Disorder Checklist for DSM-V (PCL-5)	3 months
**PTSD severity and symptoms via clinician rating**	Clinician-Administered PTSD Assessment for the Diagnostic and Statistical Manual of Mental Disorders, Fifth Edition DSM-V (CAPS-5)	3 months
**Depression**	Patient Reported Outcome Measurement Information System (PROMIS) Adult Short Form (SF) v1.0 - Emotional Distress - Depression 8a	3 months
**Anxiety**	Patient Reported Outcome Measurement Information System (PROMIS) Adult Short Form (SF) v1.0 - Emotional Distress - Anxiety 8a	3 months

Secondary

Outcome Measure	Measure Description	Time Frame
**Social isolation**	Patient Reported Outcome Measurement Information System (PROMIS) Adult Short Form (SF) v2.0 - Social Isolation 8a	3 months
**Companionship**	Patient Reported Outcome Measurement Information System (PROMIS) Adult Short Form (SF) v2.0 - Companionship 6a	3 months
**Ability to participate in social activities**	Patient Reported Outcome Measurement Information System (PROMIS) Adult Short Form (SF) v2.0 - Ability to Participate in Social Roles and Activities 8a	3 months
**Mental health quality of life**	Veterans Rand 12-item Health Survey (VR-12) - Mental Health Component	3 months
**Psychological well-being**	Bradburn Scale of Psychological Wellbeing (BSPW)	3 months
**Life satisfaction**	Satisfaction with Life Scale (SLS)	3 months
**Resilience**	Connor-Davidson Resilience Scale (CDRS)	3 months
**Anger**	Patient Reported Outcome Measurement Information System (PROMIS) Adult Short Form (SF) v1.1 - Emotional Distress - Anger 5a	3 months
**Daily socio-emotional experiences**	Ecological momentary assessment (EMA)	3 months

Other Pre-specified

Outcome Measure	Measure Description	Time Frame
**Work and activity participation**	Work Productivity and Activity Impairment Questionnaire (WPAIQ)	3 months
**Suicidal ideation**	Columbia Suicide Severity Rating Scale (CSSRS)	3 months

**Sleep quality**	Pittsburgh Sleep Quality Assessment (PSQI)	3 months
**Sleep disturbance**	Patient Reported Outcome Measurement Information System (PROMIS) Adult Short Form (SF) v1.0 - Sleep Disturbance 8a	3 months
**Sleep actigraphy**	Actiwatch 2 (Philips Respironics)	3 months
**Salivary cortisol awakening response**	Passive drool method (Salimetrics)	3 months
**Electrodermal activity (skin conductance)**	Embrace wristband (Empatica, Inc.)	3 months

Collaborators and Investigators
Sponsor: Marguerite O'Haire

Collaborators
- Eunice Kennedy Shriver National Institute of Child Health and Human Development (NICHD)
- National Center for Complementary and Integrative Health (NCCIH)

Investigators
- Principal Investigator: Marguerite O'Haire, Ph.D.,Purdue University

Statistical Analysis Plan

Since all the outcome measures are ordinal in nature (based on questionnaire scores), cumulative probability models will be used for all outcomes. While many of these outcomes have been modeled with linear regression in past studies, statisticians have cautioned that treating ordinal data as though they were continuous could potentially lead to misleading results, and that even continuous data can be modeled with cumulative probability models to avoid assumptions about the conditional distribution of the outcome (1,2). Cumulative probability models enable estimation of effect ratios (e.g., veterans allocated service dogs had a lower odds of greater CAPS-5 score compared to veterans on the waiting list), exceedance probabilities (e.g. Prob[PHQ-9 ≥ 10 | X=service dog]), and differences in exceedance probabilities (e.g. absolute risk reduction between veterans allocated a service dog vs control).

In addition to a binary variable for intervention (1=service dog, 0=control), covariates will include the baseline value of the modeled outcome, age, gender/sex, concurrent evidence-based treatment for PTSD, pet ownership, military sexual trauma, and traumatic brain injury.

We will conduct sensitivity analyses, modeling the primary outcomes with ordinary least squares regression to see if our conclusions are sensitive to the model choice, by comparing the estimated conditional means and 95% confidence intervals.

In exploratory analyses, we will consider a potential interaction between treatment and baseline PTSD severity, as baseline severity could modify the effect of service dog.
Multiple imputation will be used for missing covariate data and missing data at the 3-month follow-up visit.

Software

R package rms (orm function) or rmsb package (blrm function)

Reporting

1. Estimated conditional median (or mean) (e.g., conditional mean PCL-5 given service dog, and given no service dog), with nonparametric bootstrap 95% confidence intervals; adjusted for pre-specified covariates.
2. Estimated parameter comparing service dog group to no service dog group (e.g. odds ratio for logit link) with nonparametric bootstrap 95% confidence interval; adjusted for pre-specified covariates.
3. For established clinically important cutoffs, exceedance probabilities (Prob[Y ≥ y | X]) will be reported (e.g., Prob[PHQ-9 ≥ 10 | X=service dog]), and differences in exceedance probabilities with nonparametric bootstrap 95% confidence intervals; adjusted for pre-specified covariates.

References

1. Liddell, T. M., & Kruschke, J. K. (2018). Analyzing ordinal data with metric models: What could possibly go wrong?. *Journal of Experimental Social Psychology*, *79*, 328-348.

2. Liu, Q., Shepherd, B. E., Li, C., & Harrell Jr, F. E. (2017). Modeling continuous response variables using ordinal regression. *Statistics in medicine*, *36*(27), 4316-4335.
