# Supplementary material for: Service Dogs for Veterans and Military Members With Posttraumatic Stress Disorder: A Nonrandomized Controlled Trial
Source: JAMA Netw Open. 2024 Jun 4;7(6):e2414686. doi: 10.1001/jamanetworkopen.2024.14686 (PMC11151141; doi:10.1001/jamanetworkopen.2024.14686)
Supplement: Supplement 2. — eTable 1. Fidelity Checklist for Research on Assistance Dogs (F-RAD) eTable 2. Cohen’s d and Linear Regression Sensitivity Analysis eTable 3. Per Protocol Analysis: Primary Outcomes at 3 Months eFigure. Primary Outcomes: Exceedance Probability Distributions, Conditional on Service Dog vs Control Group eTable 4. Self-Reported Adverse Events (AE) and Serious Adverse Events (SAE) eAppendix. Columbia Suicide Severity Rating Scale (C-SSRS) and Patient Health Questionnaire (PHQ-9) Data eTable 5. Participant C-SSRS Data at Baseline and 3-Month Follow-Up eTable 6. Participant PHQ-9 Item 9 Data at Baseline and 3-Month Follow-Up eTable 7. PHQ-9 Depression at 3 Months eReferences [file jamanetwopen-e2414686-s002.pdf]

Supplemental Online Content

Leighton SC, Rodriguez KE, Jensen CL, et al. Service dogs for veterans and military members with posttraumatic stress disorder: a nonrandomized controlled trial. *JAMA Netw Open*. 2024;7(6):e2414686. doi:10.1001/jamanetworkopen.2024.14686

eTable 1. Fidelity Checklist for Research on Assistance Dogs (F-RAD)

eTable 2. Cohen's *d* and Linear Regression Sensitivity Analysis

eTable 3. Per Protocol Analysis: Primary Outcomes at 3 Months

eFigure. Primary Outcomes: Exceedance Probability Distributions, Conditional on Service Dog vs Control Group

eTable 4. Self-Reported Adverse Events (AE) and Serious Adverse Events (SAE)

eAppendix. Columbia Suicide Severity Rating Scale (C-SSRS) and Patient Health Questionnaire (PHQ-9) Data

eTable 5. Participant C-SSRS Data at Baseline and 3-Month Follow-Up

eTable 6. Participant PHQ-9 Item 9 Data at Baseline and 3-Month Follow-Up

eTable 7. PHQ-9 Depression at 3 Months

eReferences

This supplemental material has been provided by the authors to give readers additional information about their work.

© 2024 Leighton SC et al. *JAMA Network Open*

eTable 1. Fidelity Checklist for Research on Assistance Dogs (F-RAD)[a]

**(1) Intervention Design**	
Provide information on:	
✓	Theoretical model on why dogs address population needs
✓	Service dog provider name
✓	Disability-specific trained tasks (sample or full list)
✓	Handler-service dog matching process
✓	Duration and curriculum of handler-service dog partnership training
✓	Post-partnering support protocols for handler-service dog teams
✓	Description of comparison condition (e.g., dose & key components)
✓	Potential confounders identified
**(2) Service Dog Provider Training**	
Description and specification of:	
✓	Dog provider credentials (e.g., ADI accredited)
✓	Dog sources, breeds, selection, screening, training, and testing
**(3) Intervention Delivery**	
✓	Content of handler training delivered as specified
✓	Dose of handler training delivered as specified (e.g., duration)
**(4) Intervention Receipt**	
✓	Passing assessment of handler comprehension of theoretical content
✓	Passing assessment of handler-service dog team (e.g., Public Access Test)
**(5) Intervention Enactment**	
✓	Usage of service dog trained tasks
✓	Ongoing training by handler with dog

[a] The F-RAD was developed according to the guidelines set by the National Institutes of Health (NIH) Behavior Change Consortium (BCC) Treatment Fidelity Workgroup. Metrics for intervention delivery and receipt were reported by the provider organization. Metrics for intervention enactment were self-reported by participants using ecological momentary assessment and survey methods.

Handler refers to the recipient of the service dog, who in this study is the veteran.

© 2024 Leighton SC et al. *JAMA Network Open*

eTable 2. Cohen's *d* and linear regression sensitivity analysis

Outcome	Cohen's *d* (95% CI)	Difference in group means (95% CI)	P Value
**Primary Outcomes**			
PTSD			
PCL-5	-0.60 (-0.97, -0.24)	-11.1 (-15.7, -6.5)	<.001
CAPS-5	-0.66 (-0.99, -0.28)	-7.6 (-10.7, -4.4)	<.001
Depression and Anxiety			
PROMIS Depression	-0.29 (-0.63, 0.07)	-3.7 (-6.4, -0.9)	.004
PROMIS Anxiety	-0.62 (-0.99, -0.28)	-4.6 (-6.8, -2.3)	<.001
**Secondary Outcomes**			
Social Health			
PROMIS Social Isolation	-0.27 (-0.62, 0.08)	-4.3 (-7.1, -1.4)	<.001
PROMIS Companionship	0.34 (-0.04, 0.66)	4.2 (1.6, 6.8)	<.001
PROMIS Social Activity	-0.61 (-0.96, -0.24)	-4.5 (-6.9, -2.2)	<.001
Quality of Life			
BSPW	0.66 (0.28, 1.02)	2.0 (1.2, 2.8)	<.001
Positive affect	0.50 (0.14, 0.85)	1.0 (0.4, 1.5)	<.001
Negative affect	-0.64 (-1.01, -0.27)	-0.9 (-1.3, -0.5)	<.001
SWLS	0.63 (0.25, 1.02)	4.2 (2.2, 6.1)	<.001
CD-RISC-10	0.13 (-0.22, 0.49)	2.8 (0.7, 5.0)	.003
VR-12 Mental Component	0.68 (0.35, 1.08)	8.0 (4.6, 11.5)	<.001
PROMIS Anger	-0.28 (-0.63, 0.08)	-4.7 (-8.0, -1.4)	.003
**Other Outcomes**			
PTSD: PCL-5 Subscales			
B: Intrusion	-0.47 (-0.81, -0.15)	-2.1 (-3.5, -0.7)	.002
C: Avoidance	-0.58 (-0.92, -0.22)	-1.5 (-2.1, -0.8)	<.001
D: Cognition & Mood	-0.48 (-0.83, -0.13)	-3.4 (-5.2, -1.6)	<.001
E: Arousal & Reactivity	-0.52 (-0.85, -0.14)	-3.6 (-5.1, -2.0)	<.001
PTSD: CAPS-5 Subscales			
B: Intrusion	-0.48 (-0.83, -0.14)	-1.6 (-2.6, -0.6)	<.001
C: Avoidance	-0.74 (-1.11, -0.38)	-1.1 (-1.7, -0.6)	<.001
D: Cognition & Mood	-0.41 (-0.77, -0.06)	-2.2 (-3.6, -0.8)	<.001
E: Arousal & Reactivity	-0.55 (-0.90, -0.21)	-2.2 (-3.2, -1.2)	<.001

Abbreviations: PCL-5, PTSD Checklist for DSM-V; CAPS-5, Clinician-Administered PTSD Scale; PROMIS, Patient-Reported Outcomes Measurement Information System; Bradburn Scale of Psychological Wellbeing; SWLS, Satisfaction with Life Scale; CD-RISC-10, Connor-Davidson Resilience Scale; VR-12 MCS, Veterans Rand Health Survey Mental Component Score.

Cohen's *d* are calculated based on observed follow-up data in participants with available follow-up. The difference in means was estimated from linear regression models with adjustment for the baseline score (restricted cubic spline), age, gender identity, race, Hispanic ethnicity, pet dog, military sexual trauma, traumatic brain injury, and concurrent evidence-based treatment at baseline. Linear regression models were fit following multiple imputation of missing outcome scores and missing predictor variables and included all participants.

© 2024 Leighton SC et al. *JAMA Network Open*

eTable 3. Per Protocol Analysis: Primary Outcomes at 3 Months

Outcome	Intervention		Control		Group Comparison at 3 Months		
	Baseline Mean (SD) (n = 73)	3 Months Mean (SD) (n = 70)	Baseline Mean (SD) (n = 75)	3 Months Mean (SD) (n = 67)	Difference in group means (95% CI)	Odds Ratio (95% CI)	P Value
**Primary Outcomes**							
PTSD							
PCL-5	57.3 (11.2)	42.3 (16.9)	55.7 (14.3)	51.7 (16.1)	-11.6 (-16.9, -6.4)	0.22 (0.11, 0.41)	<.001
CAPS-5	42.0 (7.4)	29.8 (10.2)	40.0 (7.0)	36.9 (10.2)	-7.9 (-11.3, -4.6)	0.17 (0.09, 0.34)	<.001
Depression and Anxiety							
PROMIS Depression	64.9 (7.9)	58.4 (9.6)	62.7 (8.4)	61.4 (8.0)	-4.2 (-7.3, -1.5)	0.35 (0.17, 0.71)	.006
PROMIS Anxiety	68.4 (5.6)	62.0 (7.4)	66.5 (5.5)	66.0 (5.4)	-5.0 (-7.4, -2.2)	0.22 (0.11, 0.46)	<.001

Abbreviations: PCL-5, PTSD Checklist for DSM-V; CAPS-5, Clinician-Administered PTSD Scale; PROMIS, Patient-Reported Outcomes Measurement Information System.

Mean (SD) values were calculated based on participants with available data. The number of participants assessed for PCL-5 at follow-up in the intervention and control groups was 70 and 67 respectively; for CAPS-5 follow-up there were 63 and 66 participants. For PROMIS Depression and Anxiety there were 62 and 65 participants in the intervention and control groups.

© 2024 Leighton SC et al. *JAMA Network Open*

eFigure. Primary Outcomes: Exceedance Probability Distributions, Conditional on Service Dog vs Control Group

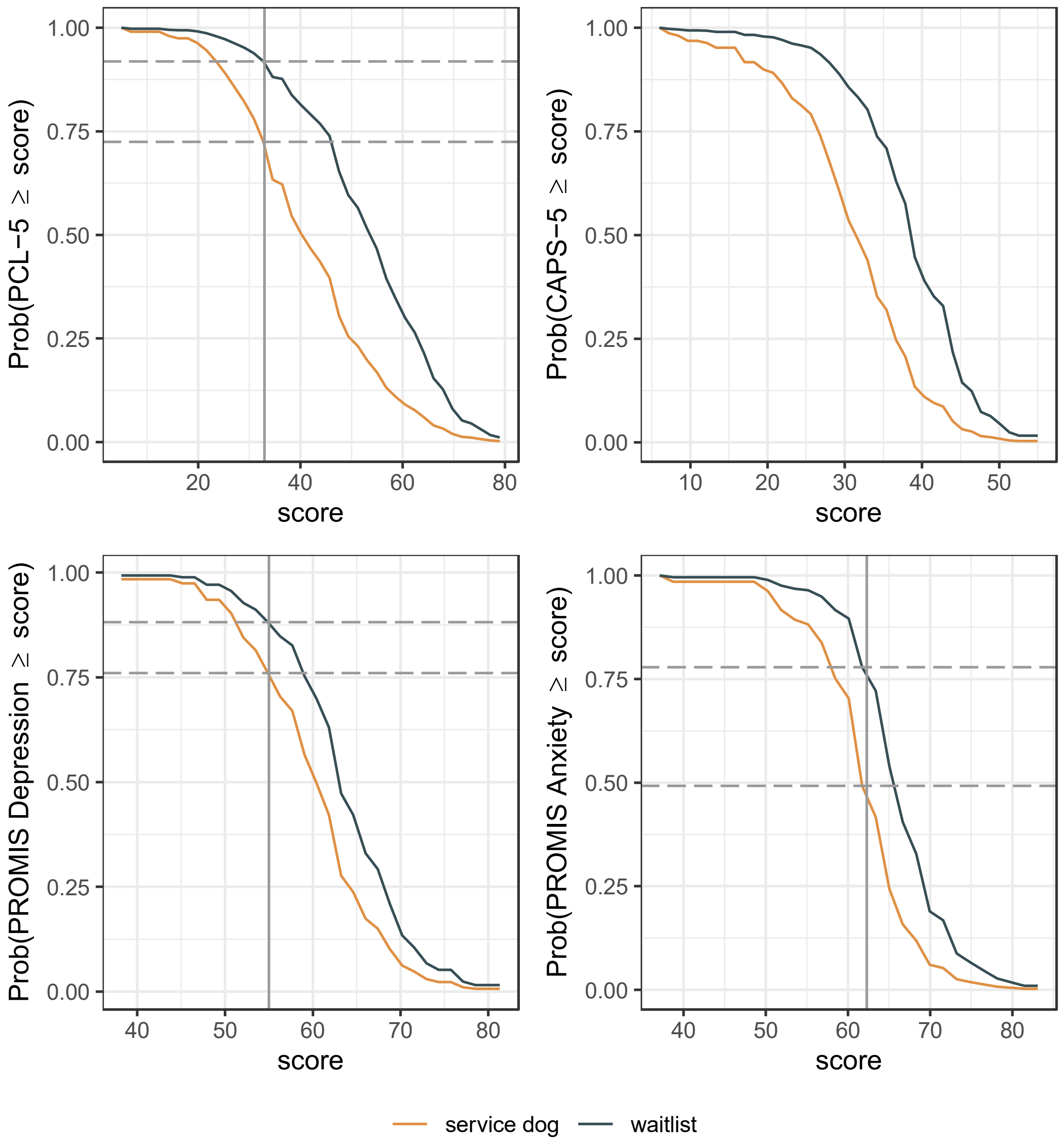

a) PTSD Checklist for DSM-5 (PCL-5): probability of provisional PTSD diagnosis (PCL-5 >= 33) at 3 months, between participants in the intervention group compared to those in the control group was 0.72 vs 0.92; absolute risk difference -0.20 (95% CI: -0.35, -0.09); b) Clinician-Administered PTSD Scale for DSM-5 (CAPS-5); c) PROMIS Depression: probability of at least mild depression (PROMIS Depression >= 55) at 3 months, between participants in the intervention group compared to those in the control group was 0.76 vs 0.88; absolute risk difference -0.12 (95% CI: -0.29, -0.02); d) PROMIS Anxiety: probability of generalized anxiety disorder (PROMIS Anxiety >= 62.3) at 3 months, between participants in the intervention group compared to those in the control group was 0.48 vs 0.78; absolute risk difference -0.30 (95% CI: -0.48, -0.12).

© 2024 Leighton SC et al. *JAMA Network Open*

eTable 4. Self-reported adverse events (AE) and serious adverse events (SAE)

Site	Relation to intervention	Intervention (N = 81)	Control (N = 75)
**AE[a]**		**1 (1%)**	**0 (0%)**
At provider site	Unrelated	0 (0%)	-
	Possibly related	1 (2%)	
	Definitely related	0 (0%)	
Not at provider site	Unrelated	0 (0%)	0 (0%)
	Possibly related	0 (0%)	0 (0%)
	Definitely related	0 (0%)	0 (0%)
**SAE[b]**		**4 (4%)**	**6 (8%)**
At provider site	Unrelated	0 (0%)	-
	Possibly related	0 (0%)	
	Definitely related	0 (0%)	
Not at provider site	Unrelated	4 (5%)	6 (8%)
	Possibly related	0 (0%)	0 (0%)
	Definitely related	0 (0%)	0 (0%)

Dash (-) indicates not applicable.

Our passive surveillance techniques allowed us insight into spontaneously reported adverse events occurring during study assessment periods (equivalent reporting opportunities across both groups) as well as during a three-week on-site period at the service dog provider (service dog group only).

[a] A total of 1 AE (1% of the service dog group) was found to be definitely related (i.e., dog bite) to the service dog intervention.

[b] A total of 10 SAEs (4% of the service dog group and 8% of the control group) occurred. Events were coded as serious adverse events (SAE) if they involved death, a life-threatening experience, inpatient hospitalization, prolongation of hospitalization, persistent or significant disability or incapacity, congenital anomaly/birth defect, or required medical, surgical, behavioral social or other intervention to prevent such an outcome. All 10 events were inpatient hospitalizations that were not related to the intervention or study (e.g., accidents, pre-existing condition treatment).

© 2024 Leighton SC et al. *JAMA Network Open*

eAppendix. Columbia Suicide Severity Rating Scale (C-SSRS) and Patient Health Questionnaire (PHQ-9) Data

The relationship between PTSD and suicidality among military veterans is complex. While PTSD is known to co-occur with and increase risk for suicidal ideation and behavior, suicidal risk is also related to a host of other biological, psychological, clinical, social, and environmental risk factors ranging from genetics to personality traits.[1,2] Notably, promotion of hope, positive future-thinking, and the presence of attainable life goals are known buffers for suicide risk.[3] These factors are all likely to be at play for a substantial number of participants in both the control and service dog groups in the present clinical trial, given that seeking partnership with a service dog is in and of itself an act of hope and a demonstration of future-thinking. Suicidality in this study was measured using the *Columbia Suicide Severity Rating Scale* (C-SSRS). In addition to enabling safety monitoring for the clinical trial, C-SSRS results are recommended to be presented in descriptive tables (eTable 5). The C-SSRS scoring and data analysis guide recommends no formal analysis, nor imputation of missing data.[4] Responses to *Patient Health Questionnaire* (PHQ-9) Item 9 (eTable 6) and Depression analysis results (eTable 7).[5]

© 2024 Leighton SC et al. *JAMA Network Open*

eTable 5. Participant C-SSRS data at baseline and 3-month follow-up

C-SSRS	Intervention (N = 81)		Control (N = 75)	
Event	Baseline	3 months	Baseline	3 months
**Suicidal Ideation Severity**				
1) Wished you were dead or wished you could go to sleep and not wake up?	44 (55%)	26 (35%)	35 (47%)	31 (46%)
Did not answer	1	6	0	8
2) Actually had any thoughts of killing yourself?	31 (39%)	12 (16%)	30 (40%)	18 (27%)
3) Been thinking about how you might do this?	17 (21%)	8 (11%)	20 (27%)	9 (13%)
4) Had these thoughts and had some intention of acting on them?	7 (8.8%)	1 (1.4%)	9 (12%)	4 (6.0%)
5) Started to work out or worked out the details of how to kill yourself?	2 (2.5%)	1 (1.4%)	2 (2.7%)	1 (1.5%)
Did not answer	1	7	0	8
**Suicidal Behavior**				
6) Prepared to do something	2 (3%)	0 (0%)	0 (0%)	0 (0%)
7) Started to do something	2 (3%)	1 (1%)	0 (0%)	0 (0%)
8) Done something	0 (0%)	0 (0%)	0 (0%)	0 (0%)
Did not answer	1	5	0	8

Abbreviations: C-SSRS, Columbia Suicide Severity Rating Scale.

© 2024 Leighton SC et al. *JAMA Network Open*

eTable 6. Participant PHQ-9 Item 9 data at baseline and 3-month follow-up

**PHQ-9 Item 9**	**Intervention (N = 81)**		**Control (N = 75)**	
Over the last 2 weeks, how often have you been bothered by thoughts that you would be better off dead, or of hurting yourself?	Baseline	3 months	Baseline	3 months
1) Not at all	41 (52%)	47 (69%)	39 (53%)	37 (57%)
2) Several days	25 (32%)	14 (21%)	23 (32%)	17 (26%)
3) More than half the days	9 (11%)	3 (4%)	7 (10%)	6 (9%)
4) Nearly every day	4 (5%)	4 (6%)	4 (6%)	5 (8%)
Did not answer	2	13	2	10

Abbreviations: PHQ-9, Patient Health Questionnaire.

© 2024 Leighton SC et al. *JAMA Network Open*

eTable 7. PHQ-9 Depression at 3 months.

Outcome	Intervention		Control		Group Comparison at 3 Months		
	Baseline M (SD) (n = 81)	3 Months M (SD) (n = 76)	Baseline M (SD) (n = 75)	3 Months M (SD) (n = 67)	Difference in group M (95% CI)	Odds Ratio (95% CI)	p
PHQ-9 Depression	16.7 (5.2)	11.6 (6.4)	16.0 (4.8)	14.5 (5.6)	-3.4 (-5.9, -1.3)	0.33 (0.17, 0.64)	.003

Abbreviations: PHQ-9, Patient Health Questionnaire. PHQ-9 ranges from 0 to 27, with lower scores indicating lower depression; clinically significant change: 5+ points.

© 2024 Leighton SC et al. *JAMA Network Open*

eReferences

1. Holliday R, Borges LM, Stearns-Yoder KA, Hoffberg AS, Brenner LA, Monteith LL. Posttraumatic stress disorder, suicidal ideation, and suicidal self-directed violence among U.S. military personnel and veterans: a systematic review of the literature from 2010 to 2018. *Front Psychol*. 2020;11. doi:10.3389/fpsyg.2020.01998

2. Turecki G, Brent DA, Gunnell D, et al. Suicide and suicide risk. *Nat Rev Dis Primer*. 2019;5(1):1-22. doi:10.1038/s41572-019-0121-0

3. Grewal PK, Porter JE. Hope theory: a framework for understanding suicidal action. *Death Stud*. 2007;31(2):131-154. doi:10.1080/07481180601100491

4. Nilsson ME, Suryawanshi S, Gassmann-Mayer C, Dubrava S, McSorley P, Jiang K. Columbia-Suicide Severity Rating Scale scoring and data analysis guide. Published online February 2013. Accessed November 1, 2023. https://cssrs.columbia.edu/wp-content/uploads/ScoringandDataAnalysisGuide-for-Clinical-Trials-1.pdf

5. Simon GE, Rutter CM, Peterson D, et al. Does response on the PHQ-9 depression questionnaire predict subsequent suicide attempt or suicide death? *Psychiatr Serv*. 2013;64(12):1195-1202. doi:10.1176/appi.ps.201200587

© 2024 Leighton SC et al. *JAMA Network Open*
