# Supplementary material for: Service Dogs for Veterans and Military Members With Posttraumatic Stress Disorder: A Nonrandomized Controlled Trial
Source: JAMA Netw Open. 2024 Jun 4;7(6):e2414686. doi: 10.1001/jamanetworkopen.2024.14686 (PMC11151141; doi:10.1001/jamanetworkopen.2024.14686)
Supplement: Supplement 3. — Data Sharing Statement [file jamanetwopen-e2414686-s003.pdf]

Data Sharing Statement

Leighton. Service Dogs for Veterans and Military Members With Posttraumatic Stress Disorder. *JAMA Netw Open*. Published June 04, 2024. doi:10.1001/jamanetworkopen.2024.14686

Data
Data available: Yes
Data types: Deidentified participant data
How to access data: maggieohaire@arizona.edu
When available: With publication

Supporting Documents
Document types: Statistical/analytic code, Informed consent form, Other (please specify)
Additional Information: Study protocol (Supplement 1)
How to access documents: maggieohaire@arizona.edu
When available: With publication

Additional Information
Who can access the data: Deidentified data are available upon reasonable request by researchers whose proposed use of the data has been approved.
Types of analyses: Specified purpose.
Mechanisms of data availability: With approved proposal and signed data access agreement.
